# Serial visual reversal learning in harbor seals (*Phoca vitulina*)

**DOI:** 10.1007/s10071-022-01653-1

**Published:** 2022-07-21

**Authors:** Nicola Erdsack, Guido Dehnhardt, Frederike D. Hanke

**Affiliations:** 1 Institute for Biosciences, Sensory and Cognitive Ecology, University of Rostock, Albert-Einstein-Strasse 3, 18059 Rostock, Germany; 2 Manatee Research Program, Mote Marine Laboratory & Aquarium, 1600 Ken Thompson Pkwy, Sarasota, FL 34236 USA; 3 Institute for Biosciences, Neuroethology, University of Rostock, Albert-Einstein-Str. 3, 18059 Rostock, Germany

**Keywords:** Cognition, Visual discrimination, Progressive improvement, Behavioral flexibility, Pinniped

## Abstract

Progressively improving performance in a serial reversal learning (SRL) test has been associated with higher cognitive abilities and has served as a measure for cognitive/behavioral flexibility. Although the cognitive and sensory abilities of marine mammals have been subject of extensive investigation, and numerous vertebrate and invertebrate species were tested, SRL studies in aquatic mammals are sparse. Particularly in pinnipeds, a high degree of behavioral flexibility seems probable as they face a highly variable environment in air and underwater. Thus, we tested four harbor seals in a visual two-alternative forced-choice discrimination task and its subsequent reversals. We found significant individual differences in performance. One individual was able to solve 37 reversals showing progressive improvement of performance with a minimum of 6 errors in reversal 33. Two seals mastered two reversals, while one animal had difficulties in learning the discrimination task and failed to complete a single reversal. In conclusion, harbor seals can master an SRL experiment; however, the performance is inferior to results obtained in other vertebrates in comparable tasks. Future experiments will need to assess whether factors such as the modality addressed in the experiment have an influence on reversal learning performance or whether indeed, during evolution, behavioral flexibility has not specifically been favored in harbor seals.

## Introduction

Cognition includes processes such as (sensory) perception, learning, and memory, as well as decision making, and problem solving (Shettleworth 2010). The cognitive and sensory abilities of pinnipeds have been subject of extensive investigation (Cook et al. 2021; Hanke and Reichmuth 2022). The numerous studies conducted so far have documented that, for example, harbor seals can easily learn demanding tasks, such as to classify novel stimulus pairs as either “same” or “different” (Scholtyssek et al. 2013). In addition to effectively dealing with novel stimuli, it might occasionally be important to change ones behavior in respect to familiar stimuli that were already associated with a specific behavior. Flexibility in behavior is particularly important for animals such as pinnipeds that inhabit highly variable environments and consequently need to effectively cope with or adapt to changes to optimize behaviors, avoid long learning phases, and, ultimately, to survive.

A classic paradigm to examine behavioral flexibility is to analyze an animal’s performance in a serial reversal learning (SRL) experiment. At the beginning of a reversal learning (RL) experiment, the subject has to learn a discrimination task with one stimulus being defined as the positive stimulus (S +), whose choice is reinforced, whereas the choice of the second stimulus, defined as negative stimulus (S−), is not reinforced. The discrimination task can either require the animal to choose one location over another, such as when choosing one arm in a Y- or T-maze in a spatial experiment (Langbein 2012; Smart 1976; Van der Borght et al. 2007), or to discriminate between two stimuli of any sensory modality, such as to discriminate two visual stimuli. After the subject has learned the discrimination, the reinforcement contingencies are reversed, meaning that now the choice of the previous S− is rewarded, whereas the choice of the previous S+ is not reinforced anymore. In SRL tasks, S+ and S− are switched every time the predefined criterion of performance is reached. It typically takes the animal longer to learn the first reversal (R1) than to learn the initial discrimination task (R0). However, in subsequent reversals (R2-Rn), many animals decrease the number of errors (Mackintosh et al. 1985; Pubols 1957; Rayburn-Reeves et al. 2013). This phenomenon is called progressive improvement. Thus, the animal is learning to learn (Harlow 1949), which is usually associated with higher levels of cognitive abilities.

While most species, even invertebrates, such as crabs (Datta et al. 1960) or spiders (Punzo 2002), seem to be able to master spatial SRL tasks, some of them even showing progressive improvement of performance (Morrow and Smithson 1969), only a few of the tested species were found to be able to master SRL tasks based on other than spatial discriminations, such as visual, olfactory, or haptic discriminations. Among these few species, progressive improvement of performance has been reported in, for example, chimpanzees (Schusterman 1962, 1964) or pigeons (Ploog and Williams 2010; Rayburn-Reeves et al. 2013).

SRL studies in marine mammals in general and in pinnipeds in particular are scarce. Bottlenose dolphins (*Tursiops truncatus*) and California sea lions (*Zalophus californianus*) were found to successfully master spatial SRL tasks with progressive improvement (Beach et al. 1974). Yaman et al. (2012) report that a bottlenose dolphin was able to master a single reversal (R) in a visual discrimination of the previously learned concept of “few” versus “many”. Schusterman (1965, 1966, 1967) tested California sea lions successfully in visual SRL experiments. However, he added a secondary cue, a previously trained size cue to the actual shape discrimination; i.e. the S+ was bigger than the S−. This way, the sea lions were able to perform the Rs without errors and, thus, showed the formation of an RL set. In a conditional RL experiment, harp seals (*Pagophilus groenlandicus*) solved a visual discrimination and successive Rs significantly faster by means of a conditional cue, in this case the testing location, than without this cue (Walsh et al. 2007). However, in neither condition, with or without conditional cue, did the seals show progressive improvement.

In this study, we set out to examine whether harbor seals, our model species for sensory and cognitive research, are able to form an RL set. Thus, we tested four harbor seals in a visual two-alternative forced-choice discrimination task and its successive Rs.

## Materials and methods

### Experimental animals and holding/testing facility

We tested four male harbor seals: Sam 19 years old, Nick 14 years old, Luca 11 years old, and Moe 7 years old when the experiment was conducted. The seals’ experimental experience varied, but none of the seals had experience in RL tasks. Seal Sam had previous experience with gratings (Hanke and Dehnhardt 2009; Weiffen et al. 2006). In general, he and Nick had already participated in numerous scientific experiments involving stimuli of different modalities (see for example Bodson et al. 2007; Kowalewsky et al. 2006; Schulte-Pelkum et al. 2007; Wieskotten et al. 2010). Luca had participated in many visual experiments (Scholtyssek et al. 2008, 2015, 2013), whereas Moe’s experimental experience was limited to hydrodynamic experiments (Krüger et al. 2018).

All animals were born and raised in captivity and held in a male-only group of seals and a fur seal. The daily routine included husbandry and medical training as well as training for behavioral experiments. The animals were held outdoors in the net-enclosure (60 m × 30 m × 2–6 m depth) of the Marine Science Center of the University of Rostock, Germany. Testing took place in a small enclosure within the large net-enclosure.

Harbor seals undergo significant seasonal metabolic changes and variations in body weight. Every seal had an individually assigned amount of food per day correspondent to its body weight and time of the year. Due to the long study period of up to one year, the daily food amount varied throughout the study period.

### Stimuli

The optic stimuli were a horizontal and a vertical black bar (6 × 20 cm) on a white background. Similar rectangles had previously been used to assess RL performance in other animals (see for example Mackintosh 1963; Mackintosh and Mackintosh 1963).

**Fig. 1:**
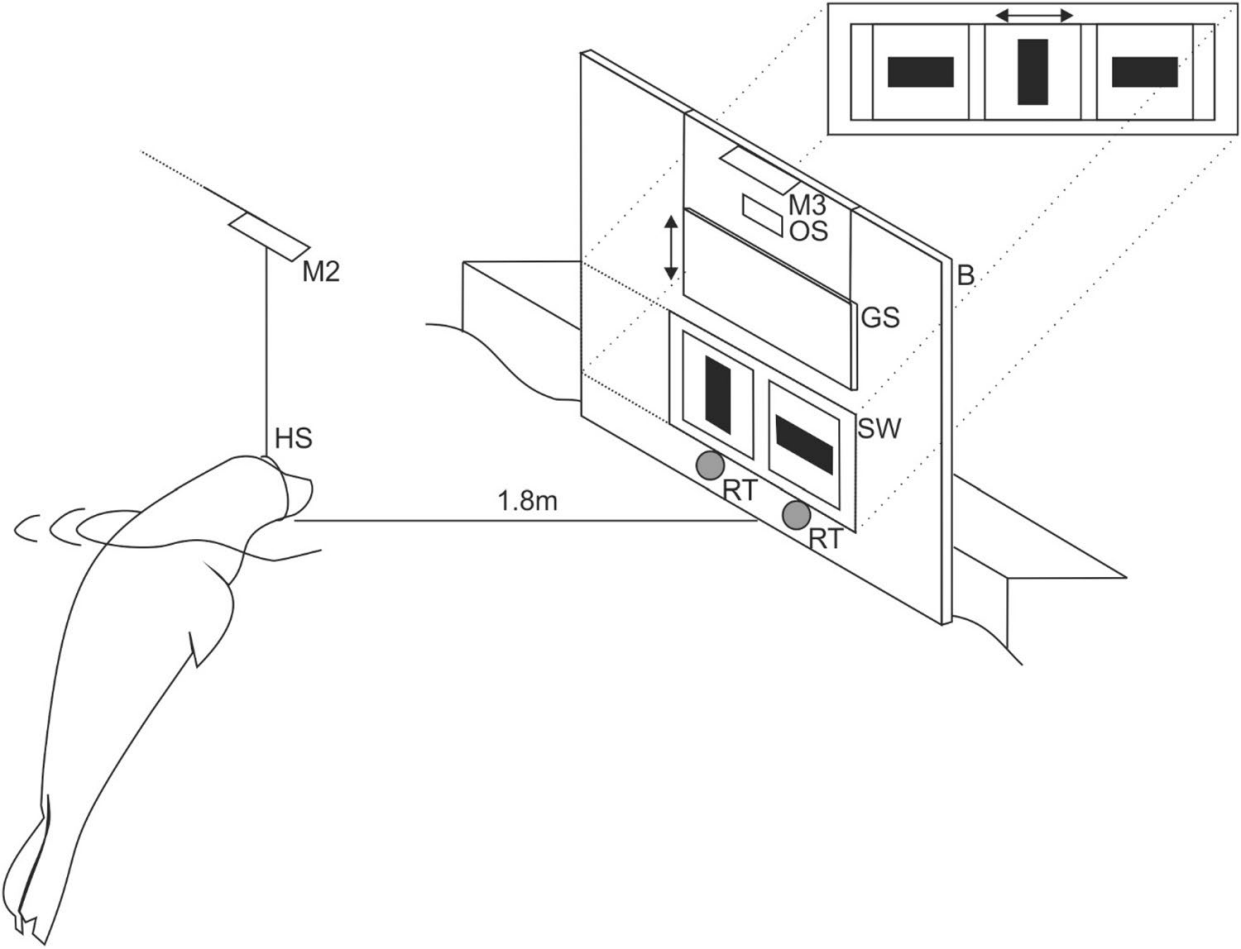
Experimental setup. The stimuli, a horizontal versus a vertical black bar (6 × 20 cm) on a white PVC board (B, 2 × 1.8 m) 15 cm above the water surface were presented to the animal stationing in a hoop station (HS) at 1.8 m distance with its head above the water surface. Between trials, stimuli were covered by a guillotine slide shutter (GS). The start of a trial was signaled by lifting the shutter upon which the animal left the station, swam towards the stimuli, and indicated its decision by touching the response target (RT) below the stimulus of its choice with the snout. Upon touching a response target, the response was recorded, and the shutter was closed. The experimenter observed the animal from behind the board through an observation slit (OS) and by means of three mirrors, M2, M3 and one mirror mounted behind the animal (not shown in the figure) to view the animal in the hoop station. Three stimuli (*S*_*x*_, *S*_*y*_, *S*_*x*_) were mounted on the sliding board (SB, shown enlarged in the inset, not drawn to scale). During the intertrial interval (ITI), the stimulus pair in the stimulus window (SW) was changed (*S*_*x*_, *S*_*y*_ or *S*_*y*_, *S*_*x*_) by moving the board to the left or right behind the closed shutter

The stimuli were presented at a distance of 27 cm from each other. The seals viewed the stimuli from a distance of 1.8 m (Fig. 1); thus, the stimuli extended over a visual angle of 1.9° × 6.3°. For all animals, the vertical bar was the S+ during the acquisition phase (R0).

### Experimental setup and procedure

The animals were tested in a visual simultaneous two-alternative forced-choice task. The experimental setup is displayed in Fig. 1. Stimuli were presented on a white PVC board (2 × 1.8 m) approximately 15 cm above the water surface. At the beginning of a trial, the animal was stationing in a hoop station with the head above the water surface. Upon lifting the guillotine slide shutter which covered the stimuli during the intertrial interval, the animal was supposed to leave the hoop station and swim towards the stimuli. It indicated its decision by touching the response target below the stimulus of its choice with the snout. Once the animal touched a response target, its response was considered final, and the shutter was closed. A correct response to the S+ was indicated by whistling and a food reward; an incorrect response was signaled by the word “nein” (German word for “no”). After the feedback given by the experimenter, the animal returned to its station. As soon as the animal was correctly positioned in the hoop station, the next trial started, and the shutter was lifted. The intertrial interval varied between 5 and 14 s.

The experimenter operated from behind the board, totally hidden from the animal’s view to exclude any secondary cues. Stimuli (*S*_*x*_, *S*_*y*_, *S*_*x*_, Fig. 1 inset, not drawn to scale) were mounted on a sliding board with only two stimuli visible to the animal at a time (*S*_*x*_, *S*_*y*_ or *S*_*y*_, *S*_*x*_). The position of the S+ was changed by moving the board sideways. To exclude auditory secondary cues, the slide board was moved sideways erratically before every trial. The experimenter was able to observe the animal’s behaviors by means of three mirrors. One mirror was mounted behind the animal (not shown in Fig. 1), which allowed viewing the animal in the hoop station in between trials. The second mirror was mounted above the hoop station, which allowed observing the animal’s response at the response targets from a distance, whereas the third mirror was installed on top of the PVC board above the observation slit and allowed a close-up view of the animal’s response behavior at the targets from above.

Usually, one to three sessions were conducted per day with variable time intervals between the sessions, five to seven days a week. Each session consisted of 30 trials with stimuli presented in orders of alternating stimuli according to Gellermann (1933). No correction trials were performed. In a session of 30 trials, a performance different from chance level (significance level) is reached with 70% correct choices. The learning criterion was defined as a performance of ≥ 80% correct choices within one session, thus clearly above significance level. Once the learning criterion was reached, the next R started in the following session. In case the learning criterion was reached in the first session of the day, the second session of the day was skipped, and the next R was started the following day. This way, a new R always started in the first session of the day. In this case (the learning criterion was reached in the first session of the day and the second session was skipped), the animal received the remaining food amount immediately after the successful session for performing other tasks not related to the experiment, such as medical training or training of new signals.

### Statistical analysis

Statistical analysis was conducted in Microsoft Excel 2010, 2014, 2019 (Microsoft, Redmond, Washington, USA). The animal’s performance during a session was documented as correct choices in percent. A chi-square test was used to assess whether the performance differed significantly from chance, corresponding to 70% correct choices in a 30-trial session. To document the performance over Rs, the total number of errors made during an R was calculated. Errors made during the session in which the animal met the learning criterion were included in the total number of errors. Data, or differences between data, respectively, were tested for normality using Shapiro–Wilk and Kolmogorov–Smirnov Tests, and for homoscedasticity by means of an *F*-Test. Performance differences between individuals, sessions, and response sides were tested for significance using two-tailed paired or homoscedastic *t*-tests, respectively. Significance level was *α* = 0.05.

## Results

**Fig. 2:**
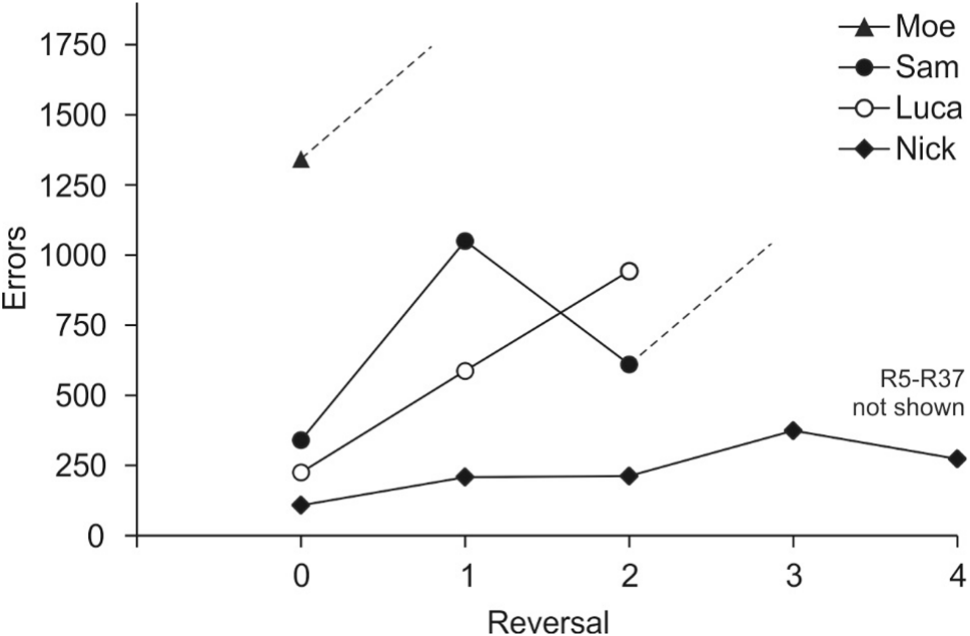
Performance of the four tested harbor seals displaying the number of errors per reversal. Please note that for seal Nick, only the first four of the 37 Rs are displayed. Dashed lines indicate that training continued after the acquisition phase in seal Moe and after R2 in seal Sam without the seals completing the respective reversal until the experiment was terminated

**Fig. 3:**
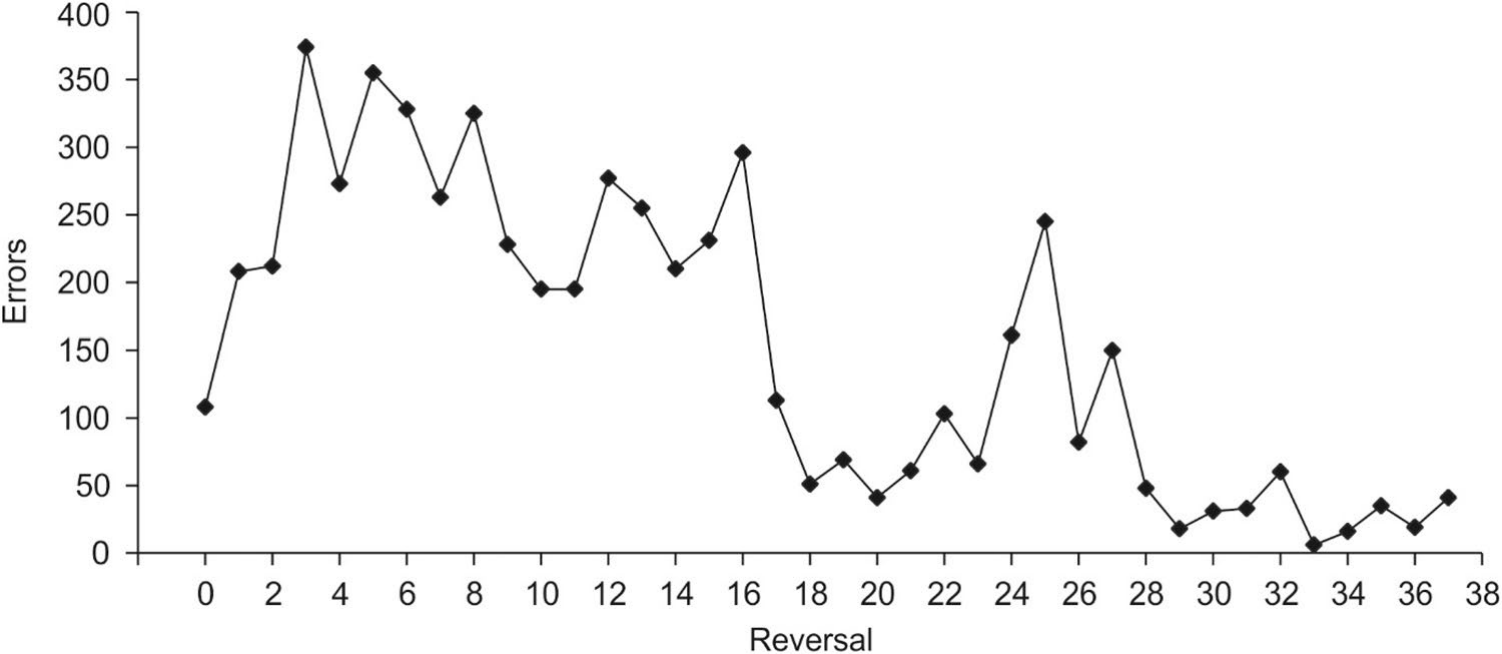
Seal Nick’s performance in number of errors per reversal. This seal successfully completed 37 reversals. His performance progressively improved to a minimum of 6 errors in R33 albeit the presence of strong fluctuations in performance

**Fig. 4:**
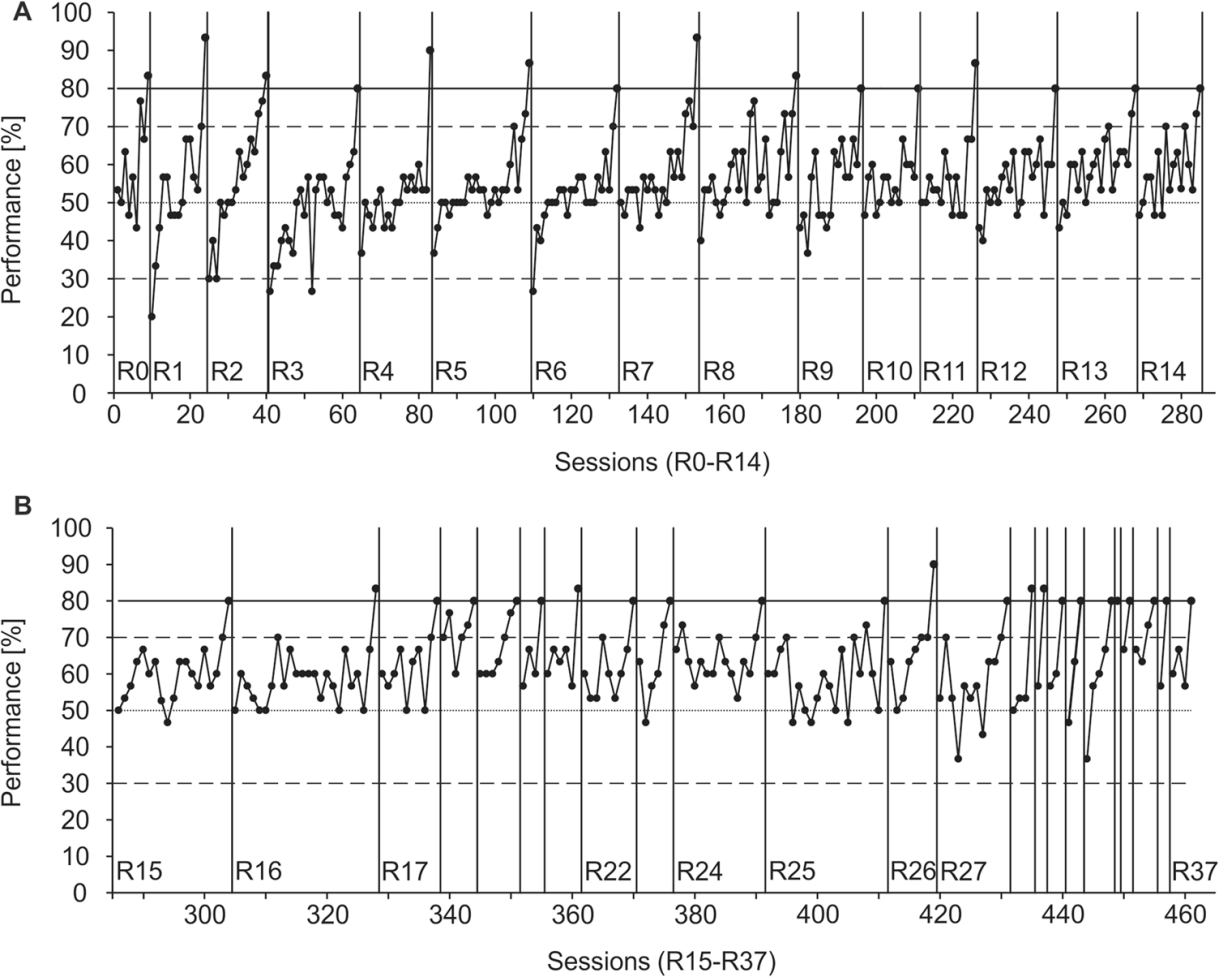
Seal Nick’s performance in % correct choices per session for **A** R0-R14, and **B** R15-R37. The solid line indicates the learning criterion of 80% correct choices to be met in one session, the dashed lines mark the upper and lower significance level of 70% and 30% correct choices, and the central dotted line at a performance of 50% corresponds to chance performance

**Fig. 5:**
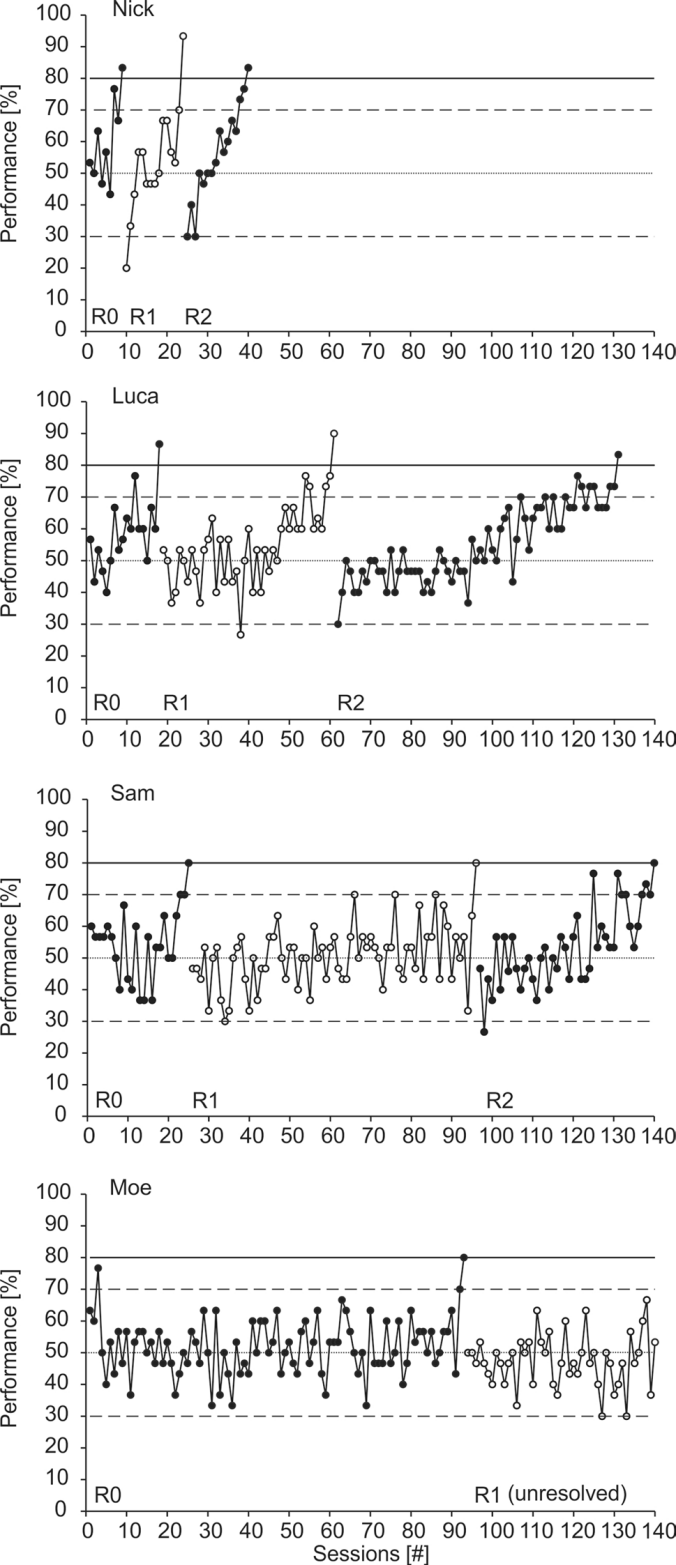
Performance in % correct choices per session for R0-R2 for all seals. For Nick, only the results of the first 2 Rs of altogether 37 Rs are displayed. Note that Moe did not complete R1, and sessions 48 to 120 of the unresolved R are not shown. The solid line indicates the learning criterion of 80% correct choices to be met in one session, the dashed lines mark the upper and lower significance level of 70% and 30% correct choices, and the central dotted line at a performance of 50% corresponds to chance performance

Performances varied immensely between individuals (Fig. 2). Although all four animals reached the learning criterion in R0, only one seal, Nick, mastered a series of 37 successive Rs; his full performance is displayed in Fig. 3 in errors per R and in Fig. 4 as % correct choices per session. In the initial discrimination, Nick reached the learning criterion with a total number of 108 errors within nine sessions, or 270 trials, respectively. This performance, as well as his performance in the first two Rs was distinctly better than the performances of the other three tested seals (Fig. 2). In all seals, performance fluctuated strongly within Rs, as shown in Fig. 5 for R0-R2. Although Nick’s performance increased progressively, meaning overall the number of errors to criterion decreased throughout the study (Fig. 3), his performance remained unstable and fluctuating throughout the entire study (Fig. 4). Not before the last ten out of the 37 Rs, Nick reached the learning criterion constantly within three sessions on average. Only once, in R33, he reached the learning criterion within one session, with a total of six errors.

On the other extreme, seal Moe did not master a single R (Figs. 2, 5). Despite performing significantly different from chance in the third session of R0, which was faster than any of the other tested seals (significant performance in Nick in session 7, in Luca in session 12, and in Sam in session 23; Fig. 5), it took him 93 sessions to reach the learning criterion in R0. However, Moe did not even reach significance level in R1. The experiment was terminated with Moe after 3600 trials (120 sessions) in R1 without any indication of performance improvement.

Seal Sam mastered two Rs (Figs. 2, 5). Although he reached significance level in eight sessions in R3 and had series of up to 13 correct choices in succession within one session, Sam did not meet the learning criterion of 80% correct choices and, thus, did not complete R3 successfully. The experiment was terminated with him after 2400 trials (80 sessions) in R3 without reaching the learning criterion. Seal Luca also mastered two Rs before he had to be pulled off the experiment for reasons unrelated to the experiment (Figs. 2, 5).

Detailed analysis of the seals’ performances revealed that Nick exhibited significantly better performances in the second session of the day in comparison to the first session (*P* << 0.001, df = 189) irrespective of the second session being conducted right after the first or with a time delay. He reached the learning criterion in the second session in 25 out of 38 cases, thus in 66% of the cases. Contrarily, the other seals’ performances did not differ significantly between the first and second session (*P* > 0.5; Moe: df = 83, Sam: df = 80, Luca: df = 59).

**Fig. 6:**
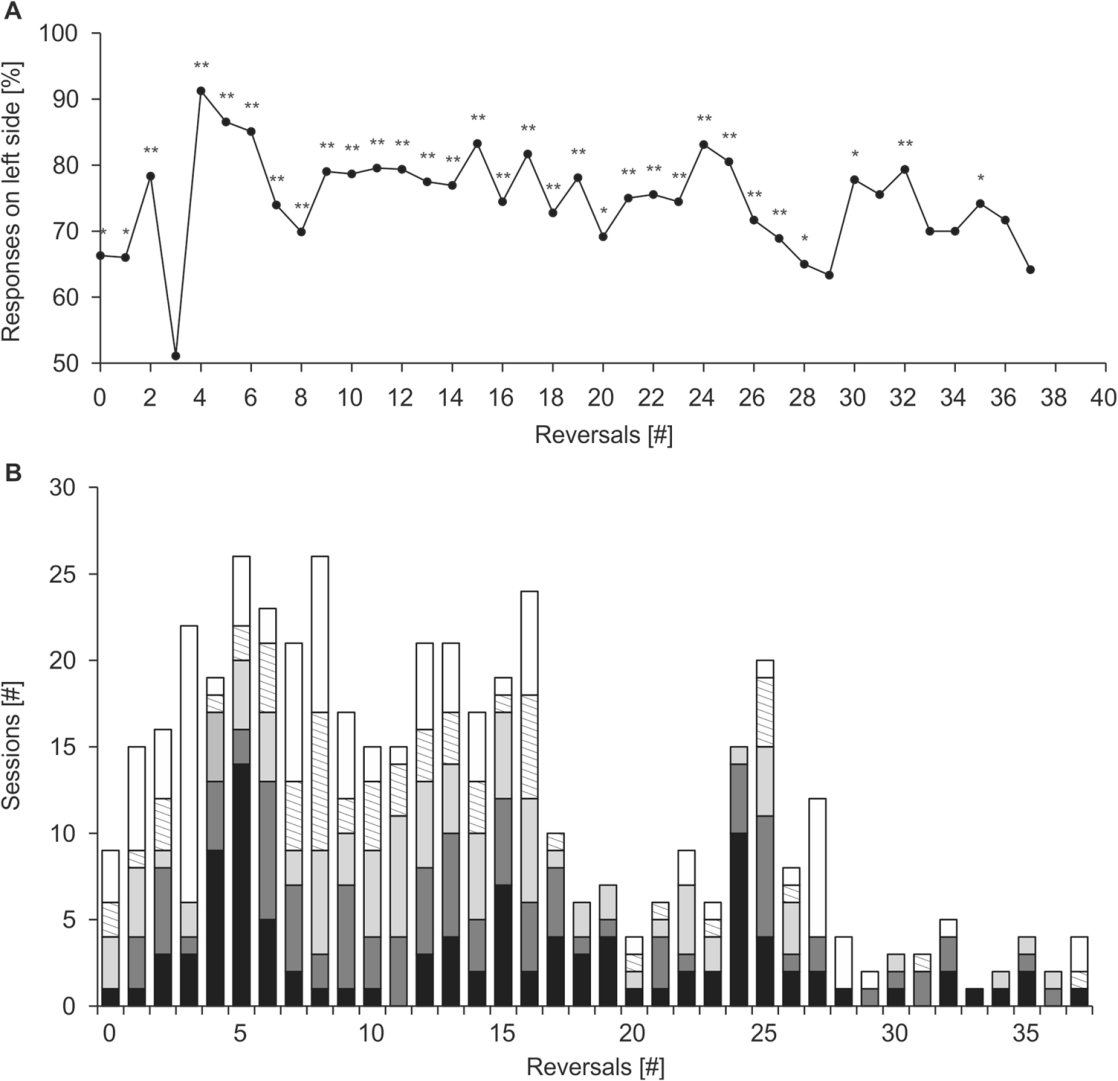
Side preference of seal Nick displayed in (**A**) % responses on the left side per R, regardless of correctness. Significant difference between % responses per side is marked with * for *P* ≤ 0.05 and ** for *P* ≤ 0.001. There is no indication for a decline in Nick's side preference before R29. **B** Absolute number of sessions per R stacked by percentage of correct responses on the left side; 100% (15 out of 15 possible correct responses, black bar), 93% (14 out of 15 possible correct responses, dark gray), 87% (13 out of 15 possible correct responses, light gray), 80% (12 out of 15 possible correct responses, shaded bar), and < 80% (less than 12 out of 15 possible correct responses, white). In 77.4% of all sessions, Nick responded correctly on the left side with performances between 80 and 100%, indicating that his success in mastering an R was determined by his correct responses when the S+ was on the right

All animals exhibited significant side preferences, as displayed in Table 1 along with the ratio of response sides in the first trials of a session. A side preference was most distinct in Nick, who responded on the left side in 76.2% of all trials (*P* << 0.001, df = 466; Fig. 6), and in 87.0% of the first trials of a session (*P* << 0.001, df = 323). Luca chose the left side in 64.2% of all responses (*P* << 0.001, df = 146), which corresponds with his responses in the first trial of a session. Moe’s preference changed throughout the study period. Overall, he responded on the left side in 54.4% of all trials (*P* = 0.001, df = 212). Only Sam exhibited a preference for the right side responding on the right side in 55.7% of all trials (*P* << 0.001, df = 219). Detailed analyses of response sides revealed that Nick's left side preference was strong from the beginning of testing and intensified from R0 to R2, while Sam exhibited a very strong right side preference in R0 (71.1%, *P* << 0.001), which turned into a left side preference in R1 (55.2%, *P* = 0.001) and back into a strong preference for the right side in R2 (66.5%, *P* << 0.001). Contrarily, Luca and Moe did not exhibit a significant side preference in R0, but developed a left side preference in R1 (Moe, incomplete: *P* << 0.001; Luca: *P* = 0.015), which intensified in Luca in R2 (*P* < < 0.001). It is noticeable that in both Sam and Moe, the preferred response side in the first trial of a session does not correspond with overall side preference.

**Table 1:** Percentage of total responses per side, significance of difference (*P*), and percentage of responses per side in the first trial of a session, listed by R and pooled for the indicated Rs

	Total responses per side [%]			First trial responses per side [%]	
	Left	Right	*P*	Left	Right
**Nick**					
R0-R37	**76.2**	**23.8**	** << 0.001**	87.0	13.0
R0-R2	**71.0**	**29.0**	** << 0.001**	78.9	21.1
R0	**66.3**	**33.7**	**0.006**	88.9	11.1
R1	**66.0**	**34.0**	**0.010**	60.0	40.0
R2	**78.3**	**21.7**	** << 0.001**	92.9	7.1
**Luca**					
R0-R2	**64.8**	**35.2**	** << 0.001**	65.4	34.6
R0	52.4	47.6	0.675	66.7	33.3
R1	**58.0**	**42.0**	**0.015**	54.1	45.9
R2	**72.1**	**27.9**	** << 0.001**	73.1	26.9
**Sam**					
R0-R2	**43.7**	**56.3**	** < 0.001**	30.0	70.0
R0	**28.9**	**71.1**	** << 0.001**	26.3	73.7
R1^b^	**55.2**	**44.8**	**0.001**	41.5	58.5
R2	**33.5**	**66.5**	** << 0.001**	15.8	84.2
**Moe**					
R0 + R1^a,b^	**54.4**	**45.6**	**0.001**	39.7	60.3
R0	47.7	52.3	0.276	26.6	73.2
R1^a^	**59.5**	**40.5**	** << 0.001**	51.1	48.9

Significant differences indicating side preferences are indicated in bold, which applies to all but Luca’s and Moe’s performances in R0

^a^Incomplete R

^b^% first trial responses opposite of side preference

In all tested animals, sessions with significantly false performance (≤ 30% correct choices) were rare exceptions. For Nick, only six sessions with significantly false performance were documented out of a total of 467 sessions. These six sessions were the initial sessions of R1, 2, 3 and 6, and another two sessions in R2 and R3. Moe performed significantly false in later sessions of R1 (sessions 34, 40, 49 and 118). Sam and Luca each performed significantly false in three, and two sessions, respectively.

## Discussion

The results of the present study show that an individual harbor seal was able to master successive Rs of a visual discrimination task with progressively improving performance. However, the results belied our expectations as only one of the four animals, Nick, was able to solve a series of Rs; the other seals mastered two Rs at maximum.

The results of this study are surprising given the fact that harbor seals had previously proven to be capable of solving complex cognitive tasks, as for example discrimination of time intervals in the millisecond range (Heinrich et al. 2016) and visual concept formation (Mauck and Dehnhardt 2005; Scholtyssek et al. 2013). Additional studies need to be conducted to unravel if there is indeed a systematic difference related to, for example, the manipulation of novel versus familiar stimuli or if variations of the RL paradigm (see discussion below) will lead to different RL results.

Although we expect individual variation within a population to be the default status as it is the substrate for evolution, we did not anticipate the extent of interindividual variation in this study. The performances of the seals ranged from an inability to complete a single R (Moe) to good SRL performance (Nick). This variation is comparable to the variation observed, for example, in the common octopus (Bublitz et al. 2017), or in horses (Sappington et al. 1997), but far stronger than the slight deviations occurring in chimpanzees or California sea lions (see for example Beach et al. 1974; Schusterman 1966). A number of factors could have an influence on the performance in an SRL study, as for example, age, previous experience, personality, and motivation. However, none of these factors can convincingly explain performance differences in our seals. First, in line with results obtained by Tapp et al. (2003) with dogs of different age, the oldest seal Sam should have shown impaired performance in comparison to the younger seals; however, Sam's performance was comparable to that of Luca. Second, there is no systematic variation of the RL performance with the degree of previous experimental experience in visual discrimination tasks, which was highest in Sam and lowest in Moe. Generally, previous experimental training could facilitate but could also interfere with subsequent learning processes. Third, the effect of the individual seal personality, defined as the sum of certain behaviors exhibited by an individual (Gosling 2001), is hard to assess. The personality or behavioral type is often correlated to a cognitive style (Sih and Del Giudice 2012), i.e., how an individual gathers and processes information, regardless of its cognitive abilities (Shettleworth 2010). A rough classification of our seals as either being of the “high speed–low accuracy”, Moe and Nick, or the “low speed–high accuracy”, Luca and Sam, behavioral type (Sih and Del Giudice 2012) did not result in a clear correlation between behavioral type and RL outcome as found for example in rats (Costanzo et al. 1975) and Florida scrub jays (*Aphelocoma coerulescens*) (Bebus et al. 2016). Fourth, motivation and cooperativeness might have affected the results of single or even series of sessions but not the overall performance as the seals did not systematically differ in their willingness to participate in the experiment. A usually high eagerness to cooperate during training and experimental testing has been achieved by adjusting the daily food amount individually for each seal, correspondent to its specific needs. This way, a consistent food motivation over time can be maintained in all seals, and we expect our seals to have been similarly motivated overall, although being fed different amounts of food.

A closer look at the performance of the most successful seal, Nick, shows that his overall performance is comparable to other species tested, as (1) he made considerably more errors during R1 in comparison to R0, and (2) he could improve his performance by reaching the learning criterion within one session in R33. However, apart from the mentioned similarities, even our best-performing seal was outperformed by representatives of other species: (1) the seal made considerably more errors during all stages of RL, (2) he did not improve his performance directly after R1, but instead his performance declined until reaching 355 errors in R5, and only in R18 the error rate dropped below the error rate of R0, and (3) he showed a slower, non-gradual progression of improvement reaching 6 errors at minimum. For comparison, goldfish tested in a color discrimination SRL task improved their performance from approximately 50 errors in R1 to approximately 25 errors in R10 (Engelhardt et al. 1973). The goldfishes' performance is still inferior to the performance of rats with approximately 30 errors in R1 and 5 errors in R10 (Mackintosh et al. 1985). Some animals even achieve one-trial learning, such as rats (Mackintosh et al. 1968) and chimpanzees (Schusterman 1964), often based on a win-stay/lose-shift strategy (Levine 1965). Thus, we do not have evidence that the seal acquired a RL set, in contrast to other species.

In contrast to pigeons performing in visual discrimination reversals (Macphail 1976; Lissek et al. 2002), stimulus perseveration seems to have played only a minor role in the seals’ performance, as indicated by the rare occurrence of sessions with significantly false performance in all seals (Fig. 5), occurring in Nick only in R1-3 and R6 (Fig. 4). In R2, Nick, Luca, and Sam returned to performance at chance level after responding significantly false in the first or second session, respectively, of the R (Fig. 5). Further indication for the minor role of stimulus perseveration was given by Nick in the rapidly decreasing number of sessions with performance < 50% correct choices from R3 to R9 (Fig. 4).

Rather than stimulus perseveration, along with their unsteady highly fluctuating performances, all four seals exhibited significant side preferences, albeit differing in consistency and intensity (Table 1). Instead of following a win-stay/loose-shift strategy, Nick’s strategy was a strong left side preference, which was apparent as early as in R0 and intensified over subsequent Rs. Besides answering predominantly on the left side in the first trial of a session, Nick’s performance included several sessions with ≥ 96.6% responses on the left side, corresponding to 29 or 30 responses on the left side in a session of 30 trials, from R2 to R7. The most extreme case occurred in R5 with 15 out of 26 sessions with ≥ 96.6% left side responses, including two series of each 7 consecutive sessions without or with only one single response on the right side. In 357 out of 461 sessions (77.4%), Nick responded correctly in 12–15 out of 15 (80–100%) possible trials with the S+ on the left side (Fig. 6b), indicating that his success in an R was predominantly determined by his correct responses on the right side. This side preference may have been the seal's response to forgetting the S+ of the previous session at the beginning of a new session; recalling the S+ gets more and more difficult the more Rs the seal has already experienced (Mackintosh 1974). However, as he did not apply a win-stay/lose-shift strategy, Nick was not able to optimize his response behavior based on his “choose left side in the first trial” strategy, neither to reach a high performance level in the subsequent second trial nor to reach one-trial learning.

In contrast, Nick’s learning curve demonstrates over the course of training that he started the Rs and continued performing at chance level, and, in 16 out of 37 Rs, his performance suddenly increased to learning criterion (Fig. 4), for example in R4 from 53.3% correct choices in session 18 to 90% in session 19, without having reached significance level before. Similar leaps could also be observed in the performances of the other seals (Fig. 5; Luca: 60–86.7% in R0; Sam: 33.3–63.3–80% in R1; Moe: 43.3–70–80% in R0). Unsteady performances and sudden performance leaps, as observed in all seals throughout the study, were described by Grether and Maslow (1937) who investigated problem solving in different monkey species and identified three main types of problem solving: (1) immediate solution, implying immediate success without learning; (2) delayed, sudden solution, describing sudden success occurring after a series of sessions at chance level; and (3) irregular improvement, describing fluctuating performances, even after reaching significance level. Problem solving types 2 and 3, fluctuating performance with sudden success after consecutive performance at chance level (Grether and Maslow 1937) match the performances of the seals in this study. These observed sudden success events might have been caused by increased motivation in these particular sessions and/or by an AHA! experience (Kaplan and Simon 1990). In Nick, the sudden solutions of the R problem were clearly discernable by behavioral changes, such as increased body tension in the hoop station and faster and more targeted response behavior. Thus, rather than acquisition of an RL set, the seal's performance seems to be best described by multiple sudden solutions of the R problem.

In contrast to Nick, Moe and Sam not only exhibited inconsistent side preferences, but in parts their preferred response side in the first trial of a session was opposed to the respective side preference during an R. Luca, who performed next best to Nick, exhibited, similar to Nick, a consistent preference for the left side throughout testing, although not significant in R0 but with a visible trend (52.4%), and consistent with his first trial behavior. The absence of a consistent side preference in the two seals with the lowest performances as opposed to the consistent side preference exhibited by the most successful and second best performing individuals is striking. It suggests that the interindividual performance differences observed in this study may have been, at least in parts due to individually differing problem solving abilities and strategies (Grether and Maslow 1937).

For the abovementioned comparisons with other species, methodological differences of the mentioned studies were neglected. Most likely, these differences affect the performance (Mackintosh et al. 1985). Generally, it would be interesting to document the seals’ performances after systematically changing certain parameters of the experimental design of our study in a future experiment. One parameter that might change the outcome of visual RL could be the duration of the intertrial interval (ITI), which was rather long in our study as after the feedback the animal needed to return to the hoop station. Previously, it was shown in pigeons that performance increases the shorter the ITI (Williams 1971, 1976) as a short ITI allows effectively using the outcome of the previous trial for the subsequent response. Thus, shortening the ITI in a future experiment could increase the performance of our seals.

Furthermore, the sensory modality addressed in a discrimination task affects the performance in RL experiments (Izquierdo et al. 2017). According to the so-called “modality specificity”, the sensory modality with which an object is perceived influences not only the perception of the object but also its representation in memory and related cognitive processes, such as categorization (Klatzky and Lederman 2001). Mackintosh et al. (1985) suggest that higher cognitive skills may be restricted to the species-specific dominant sensory modality, for example, the visual system in humans (Posner et al. 1976), or the auditory system in toothed whales (Cozzi et al. 2017). Kea (*Nestor notabilis*), for example, a New Zealand parrot species known for its manipulative skills, learned a discrimination task and a subsequent R significantly faster choosing between solid objects than between visual stimuli on a touchscreen (O'Hara et al. 2015). Comparable results were obtained in rats (Brushfield et al. 2008), which learned an olfactory discrimination and its reversal significantly faster than a visual discrimination and the corresponding R task. Future RL experiments could contrast the harbor seals’ performance in a visual SRL experiment with an SRL experiment involving stimuli of a different modality. Different results might then be obtained as, due to environmental constraints such as turbidity or darkness, harbor seals cannot always rely on their visual system. Other senses might be more dominant than vision, and asking the seals to perform in tasks involving other modalities might increase RL performance.

A very promising approach could be to contrast the seals’ performance in the current visual SRL experiment with their performance in a spatial SRL experiment (Niesterok et al. 2022); a comparison that has previously been done in other species (see for example Doty and Combs 1969). We would hypothesize that seals perform better in a spatial SRL experiment as (1) previous experiments have documented better spatial as compared to visual abilities (Mauck and Dehnhardt 2007; Renouf and Gaborko 1989), (2) a better spatial awareness can be deduced from the seals’ lifestyle, and (3) a decreased visual performance seems to mirror the fact that vision may be reduced in the seals’ environment when diving in dark or turbid water. (The data of our spatial RL experiment (Niesterok et al. 2022) are reported in this volume, too).

In conclusion, our study demonstrates that, in general, a harbor seal is able to learn multiple Rs of a visual discrimination with progressive improvement of performance. However, the seals’ performances differed significantly, with only one out of four tested seals having mastered serial reversals, but with significantly more errors, and slower, fluctuating progression of improvement than other vertebrate species in comparable tasks. Interindividual performance differences may have originated in differing abilities and strategies to solve the reversal problem. Further testing will reveal whether different task modalities may improve the seals' SRL performances, or maybe behavioral flexibility does not play a vital role in a harbor seal's life.

## Data Availability

All data are presented in the manuscript. Detailed learning performances can be obtained from the authors upon request.
